# Machine-learning and structure-based discovery of SARS-CoV-2 papain-like protease (PL^pro^) inhibitors with efficacy in a murine infection model

**DOI:** 10.1063/4.0001004

**Published:** 2025-10-27

**Authors:** Ellene H. Mashalidis

**Affiliations:** Pfizer, Groton, CT, USA

## Abstract

First-generation antiviral therapeutics have provided important protection against COVID-19 caused by severe acute respiratory syndrome coronavirus 2 (SARS-CoV-2). However, additional therapeutic mechanisms are needed that provide enhanced efficacy and protection against potential viral resistance. The SARS-CoV-2 papain-like protease (PL^pro^) is one of the two essential cysteine proteases involved in viral replication. While inhibitors of the SARS-CoV-2 main protease have demonstrated clinical efficacy, previously reported PLpro inhibitors like GRL0617 have lacked the cellular inhibitory potency to demonstrate that targeting PLpro translates to *in vivo* efficacy in a preclinical setting. Here, we report the machine learning–driven discovery of potent, selective, and orally available SARS-CoV-2 PLpro inhibitors, with lead compound PF-07957472 providing robust efficacy in a mouse-adapted model of COVID-19 infection. Structural elucidation of PF-07957472 bound to SARS-CoV-2 PL^pro^ by X-ray crystallography provides rationale for the enhanced potency observed over GRL0617. We used this structural understanding and platform as a basis to design additional potent compounds with improved off-target liabilities, such as human ether-a-go-go (hERG) and CYP3A4 time-dependent inhibition (TDI). This work demonstrates the value of integrating machine-learning methods with traditional structure-based drug design to rapidly arrive at potent antiviral compounds.

